# Direct, Differential Effects of Tamoxifen, 4-Hydroxytamoxifen, and Raloxifene on Cardiac Myocyte Contractility and Calcium Handling

**DOI:** 10.1371/journal.pone.0078768

**Published:** 2013-10-24

**Authors:** Michelle L. Asp, Joshua J. Martindale, Joseph M. Metzger

**Affiliations:** Department of Integrative Biology and Physiology, University of Minnesota Medical School, Minneapolis, Minnesota, United States of America; Temple University, United States of America

## Abstract

Tamoxifen (Tam), a selective estrogen receptor modulator, is in wide clinical use for the treatment and prevention of breast cancer. High Tam doses have been used for treatment of gliomas and cancers with multiple drug resistance, but long QT Syndrome is a side effect. Tam is also used experimentally in mice for inducible gene knockout in numerous tissues, including heart; however, the potential direct effects of Tam on cardiac myocyte mechanical function are not known. The goal of this study was to determine the direct, acute effects of Tam, its active metabolite 4-hydroxytamoxifen (4OHT), and related drug raloxifene (Ral) on isolated rat cardiac myocyte mechanical function and calcium handling. Tam decreased contraction amplitude, slowed relaxation, and decreased Ca^2+^ transient amplitude. Effects were primarily observed at 5 and 10 μM Tam, which is relevant for high dose Tam treatment in cancer patients as well as Tam-mediated gene excision in mice. Myocytes treated with 4OHT responded similarly to Tam-treated cells with regard to both contractility and calcium handling, suggesting an estrogen-receptor independent mechanism is responsible for the effects. In contrast, Ral increased contraction and Ca^2+^ transient amplitudes. At 10 μM, all drugs had a time-dependent effect to abolish cellular contraction. In conclusion, Tam, 4OHT, and Ral adversely and differentially alter cardiac myocyte contractility and Ca^2+^ handling. These findings have important implications for understanding the Tam-induced cardiomyopathy in gene excision studies and may be important for understanding effects on cardiac performance in patients undergoing high-dose Tam therapy.

## Introduction

Tamoxifen (Tam), a widely used therapeutic for the treatment and prevention of breast cancer, is a Selective Estrogen Receptor Modulator (SERM) [1]. SERMs bind to and alter estrogen receptor (ER) function by inhibiting the binding of endogenous estrogens. In addition to its use for breast cancer treatment, Tam has been studied in clinical trials for the treatment of childhood gliomas [2–5] and cancers with multiple drug resistance [6,7]. The proposed mechanisms of action for Tam in these cancers are the inhibition of PKC [8] and P-glycoprotein [9], for gliomas and cancers with multiple drug resistance, respectively. These effects are ER-independent and require high doses, achieving ~5-100 fold higher serum concentrations compared to women treated for breast cancer. In these studies, some patients developed Long-QT Syndrome (LQTS) [4,5,7], which was reversible upon lowering the Tam dose or discontinuing treatment [4,10]. Tam is known to acutely inhibit multiple ion channels in the sarcoplasmic reticulum (SR) and plasma membrane [11–13], providing a potential mechanism for the prolonged action potential duration characteristic of LQTS. Whether these Tam-induced electrophysiological changes are associated with altered contractile function at the cellular level has not been determined.

 Tam is also used in biomedical research involving experimental mouse models to probe spatiotemporal gene function in multiple tissues [14–17], including heart [18]. Transgenic expression of Cre recombinase driven by a tissue-specific promoter allows for excision of loxP-flanked genes to create a tissue-specific knockout animal. To study heart-specific gene function in mice, Cre is driven by the alpha-myosin heavy chain promoter [18]. Temporal specificity is attained through fusion of Cre to a protein with a modified estrogen receptor [18–20]. Modified estrogen receptor (Mer) contains the ligand binding domain of the murine estrogen receptor (amino acids 281-599) with a GR mutation at position 525, abolishing its estrogen-binding activity while retaining its affinity for Tam and its active metabolite 4-hydroxytamoxifen (4OHT) [21]. Tam administration in mice with a MerCreMer transgene (Tg(αMHC-MerCreMer)) causes displacement of Hsp90 proteins associated with MerCreMer [22] and reveals the nuclear localization sequence of Cre. This leads to nuclear translocation of MerCreMer, and Cre-mediated cardiac gene excision in a time-specific manner [18,20]. This technology has allowed scientists to circumvent embryonic and early postnatal lethality of cardiac gene knockdown by initiating gene excision in adult mice [23]. Additionally, inducible Cre recombinase decreases adverse effects of constitutive Cre expression on heart function [24]. 

One concern of MerCreMer-mediated gene excision is the onset of severe transient dilated cardiomyopathy after Tam treatment independent of gene excision [25]. Tam-induced cardiomyopathy in Tg(αMHC-MerCreMer) mice has been proposed to be Mer-dependent by causing an increase in nuclear Tam accumulation and subsequently altering transcription of genes related to heart function and metabolism [25]. *In vivo* hemodynamics data also suggest an independent effect of Tam to suppress cardiac function [25]. Changes in gene transcription with Tam [26], albeit relatively small compared to Tam and MCM in combination, may partially explain the observed *in vivo* effects. Acute, non-genomic effects of Tam administration have thus far not been considered as a contributor to Tam-induced dilated cardiomyopathy in Tg(αMHC-MerCreMer) mice. PKA is implicated as a non-genomic signaling target of ERs with 17β-estradiol, causing decreased phosphorylation of PKA target proteins with subsequent alterations in contraction and SR Ca^2+^ release and reuptake [27–29]. It is currently unknown whether acute ER signaling through Tam has direct effects on cardiac myocyte function.

The purpose of the present study was to determine the direct, acute, non-genomic effects of tamoxifen on cardiac myocyte mechanical function. To test these effects, isolated adult rat cardiac myocytes were acutely treated with Tam, 4OHT, or the related SERM Raloxifene (Ral), and contractility and Ca^2+^ transient data were collected. These experiments have significance for understanding the pathophysiology of high dose Tam treatment on the heart with applications for Tam-inducible gene knockout mouse models and clinical medicine. 

## Methods

### Ethics Statement

All research conformed to the statutes of the Animal Welfare Act and the guidelines of the Public Health Service as issued in the Guide for the Care and Use of Laboratory Animals. All animal procedures were approved by the University of Minnesota Institutional Animal Care and Use Committee (NIH Animal Welfare Assurance Number: A3456-01).

### Adult Rat and Mouse Cardiac Myocyte Isolation and Culture

Adult rat cardiac myocytes were isolated and cultured as previously described [30]. Briefly, female Sprague-Dawley rats (Harlan Laboratories, Inc., Indianapolis, IN), weighing approximately 200g, were given sodium heparin (1500 units/kg) and anesthetized with Nembutal (162.5 U/kg). When rats were unresponsive as tested by toe pinch reflex, hearts were excised, mounted on a modified Langendorff apparatus and retrograde-perfused with oxygenated Ca^2+^-free Krebs Henseleit buffer (118 mM NaCl, 4.8 mM KCl, 25 mM Hepes, 1.2 mM KH_2_PO_4_, 1.2 mM MgSO_4_ x 7H_2_0, 11 mM glucose) containing collagenase type II (Worthington Chemical Corp., Lakewood, NJ) at 37°C for approximately 25 min. Calcium was added back to a concentration of 0.625 mM during this time. After perfusion, the ventricles were cut into ~10 pieces, gently swirled in collagenase-containing Krebs, and minimally titurated with a large bore transfer pipet at 37°C causing individual cells to dissociate from the tissue. Cell fractions containing >70% rod-shaped cells were briefly spun in a clinical centrifuge and resuspended in Krebs containing 2% BSA where Ca^2+^ was then titrated up to 1.8 mM. Cells were plated on laminin-coated coverslips and cultured at 37°C and 5% CO_2_ in M199 medium (Gibco, Life Technologies, Grand Island, NY) containing 26 mM NaHCO_3_, 25 mM Hepes, 10 mM glutathione, 0.2% (w/v) BSA, 1% (v/v) ITS (insulin, transferrin, sodium selenite, Sigma Aldrich I1884), and 1% (v/v) Penicillin/Streptomycin. Mouse myocyte isolation was carried out similarly to the rat isolation with the following modifications: Krebs Henseleit buffer was supplemented with 10 mM 2,3-Butanedione monoxime and 30 mM Taurine, and hearts were perfused with collagenase for 10 min. 

### Treatments

All drugs were obtained from Sigma Aldrich (St. Louis, MO). Tamoxifen (T5648) was dissolved in ethanol at 50 mM, 4-hydroxytamoxifen (H6278) was dissolved in methanol at 25 mM, and Raloxifene Hydrochloride (R1402) was dissolved in DMSO at 50 mM. Stock solutions were aliquoted and stored at -20°C. 4-hydroxytamoxifen was protected from light. Before an experiment a 0.5 mM working stock was made by diluting in Modified Tyrode’s Solution (140mM NaCl, 5 mM KCl, 0.5 mM MgCl_2_, 5 mM Hepes, 5.5 mM glucose, 1.8 mM CaCl_2_, pH 7.4). Each drug was tested at multiple concentrations (0, 0.5, 1, 3, 5, and 10 µM), with the total amount of vehicle remaining constant. Because drugs precipitated out of the working stock over time, fresh working stock was prepared for each concentration tested. For each experiment, the different concentrations of drug were tested in random order. 

### Sarcomere Length Measurements

**Figure 1:**
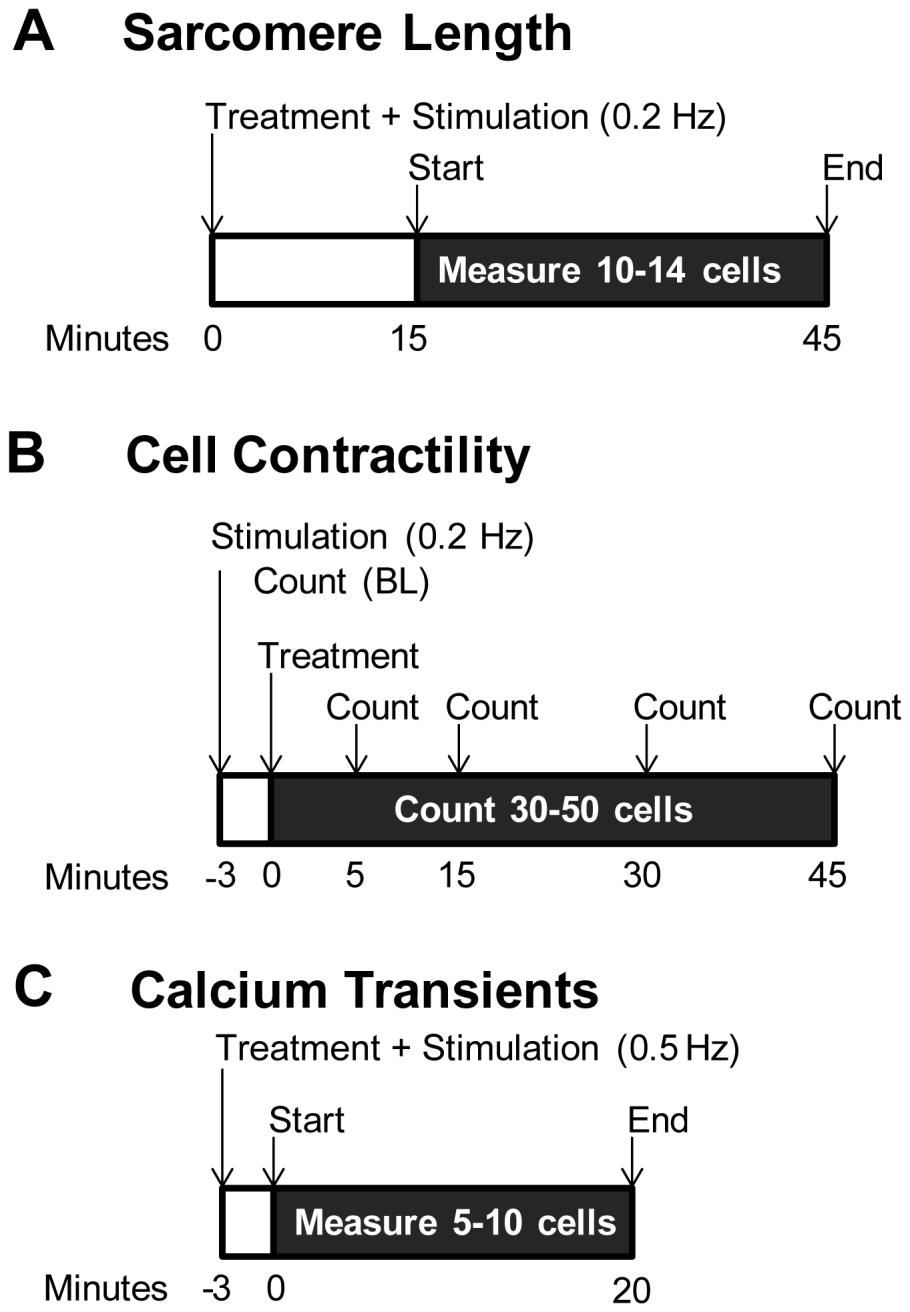
Experimental Timelines. A) For sarcomere length measurements, cells were covered in Tyrode’s containing Tam, 4OHT or Ral, allowed to reach 36 ± 1°C, and then stimulated at 0.2 Hz. Data was collected from 15-45 minutes. B) For cell contractility measurements, the number of contracting cells were counted at baseline, and 5, 15, 30 and 45 minutes after administration of the treatment. C) For Ca^2+^ transient measurements, cells were stimulated at 0.5 Hz for 3 minutes and then data was collected for 20 minutes.

Saromere length and contraction/relaxation kinetics were tested using the Myocyte Calcium and Contractility System (Ionoptix, Milton, MA) [30]. Rat myocytes were tested the day after cell isolation. Glass coverslips containing cells were mounted onto a heated stimulation chamber and covered with pre-warmed Modified Tyrode’s Solution containing the drug being tested. Cells were visualized on an inverted microscope (Nikon Eclipse TE2000-U) using a 40X objective. When cells reached 36±1°C (~2 min), they were stimulated for 15 min at 0.2 Hz and 25 V. Sarcomere length data was collected between 15 and 45 min after beginning stimulation. Cells were given fresh pre-warmed Tyrode’s with drug about every 10 min throughout the experiment. IonWizard software (Ionoptix, Milton, MA) acquires data at 250 Hz and employs Fast Fourier Transform analysis to measure sarcomere shortening kinetics. Ten or more contractions were averaged together for each cell, and ~10-14 cells were measured per treatment, evenly spaced over the 30 min data collection period (Figure 1A). Cells from three to four separate rat myocyte preparations were analyzed for each drug. Mouse myocyte sarcomere length experiments were carried out the same day they were isolated. Cells were treated with 10 µM Tam or Ral, and data was collected from 5-30 min after treatment. Mouse myocytes from two separate cell preparations were analyzed.

### Myocyte Contractility

Observationally, there was a time and dose dependent effect for rod-shaped cells to stop contracting. Thus, measuring changes in sarcomere length alone underestimates the total effect of the drug. To quantify the number of myocytes that stopped contracting in response to field stimulation, rod-shaped cells that were visibly contracting or not contracting were counted in a single field of view before and 5, 15, 30, and 45 min after drug treatment. Myocytes were viewed with a 10X objective, and approximately 30-50 myocytes were counted during each experiment (Figure 1B). The percentage change from baseline in the number of contracting myocytes for each time point was used for analysis. 

### Calcium Handling

Calcium transients were also measured using the Ionoptix Myocyte Calcium and Contractility System (Milton, MA) [30]. The system employs the interpolated numerator method where, once every 10 sec throughout the data collection period, the filter switches to 360 nm, the isobestic point. During the remainder of the time, data is collected at 380 nm, which is the wavelength of absorption for Ca^2+^ free Fura-2AM. The 360:380 ratio is used as a relative measure of cytosolic Ca^2+^ concentration. Before the experiment, cells were loaded with 2 μM Fura-2AM (Molecular Probes, Life Technologies, Grand Island, NY) for 15 min and then de-esterification proceeded for 10 minutes. Myocytes were mounted onto the heated stimulation chamber and covered with pre-warmed Tyrode’s Solution containing the treatment. After reaching 36±1°C (~2 min), myocytes were stimulated at 0.5Hz and 25V for ~3 min and transients from 5-10 cells were collected over the next 20 min (Figure 1C). Ten or more transients were averaged together for each myocyte. Background fluorescence was subtracted from the numerator and denominator by collecting a few seconds of data in an empty field of view in close proximity to the cell measured. Three to four separate rat myocyte preparations were analyzed for each drug.

### Isoproterenol Rescue Experiment and Western Blots

Cells were treated with one of the following: 1) vehicle for 15 min, 2) 10 nM isoprenaline hydrochloride (Iso, 15627 Sigma Aldrich) for 15 min, 3) 10 μM Ral for 15 min, 4) Iso for 5 min then Iso + Ral for 10 min, 5) Ral for 5 min then Ral + Iso for 10 min. After 15 min, cells were pelleted and resuspended in RIPA buffer with 0.5% SDS and protease/phosphatase inhibitors. Samples were boiled with 4x Laemmli buffer, and 20 μg protein was run on a 4-12% Bis-Tris gel. Protein was transferred to a PVDF membrane and blotted with anti-phospho-cTnI (4004, Cell Signaling Technologies, Danvers, MA) and anti-actin (A2103, Sigma Aldrich) antibodies. Bands were visualized using the Infrared Odyssey Imaging System (LI-COR, Lincoln, NE). Because there were no differences between groups 4 and 5, the data from these groups were combined for statistical analysis. 

### Statistical Analysis

Sarcomere length, Ca^2+^ transient data, and Western blots were analyzed by one-way ANOVA with Dunnett’s test for multiple comparisons, comparing each concentration of drug to the control (0 μM). Contractility experiments were analyzed by repeated measures ANOVA and Bonferroni post-hoc test for multiple comparisons. P < 0.05 was considered statistically significant. All analyses were done using GraphPad Prism 5 (GraphPad Software, Inc., La Jolla, CA). 

## Results

### Tamoxifen

**Figure 2:**
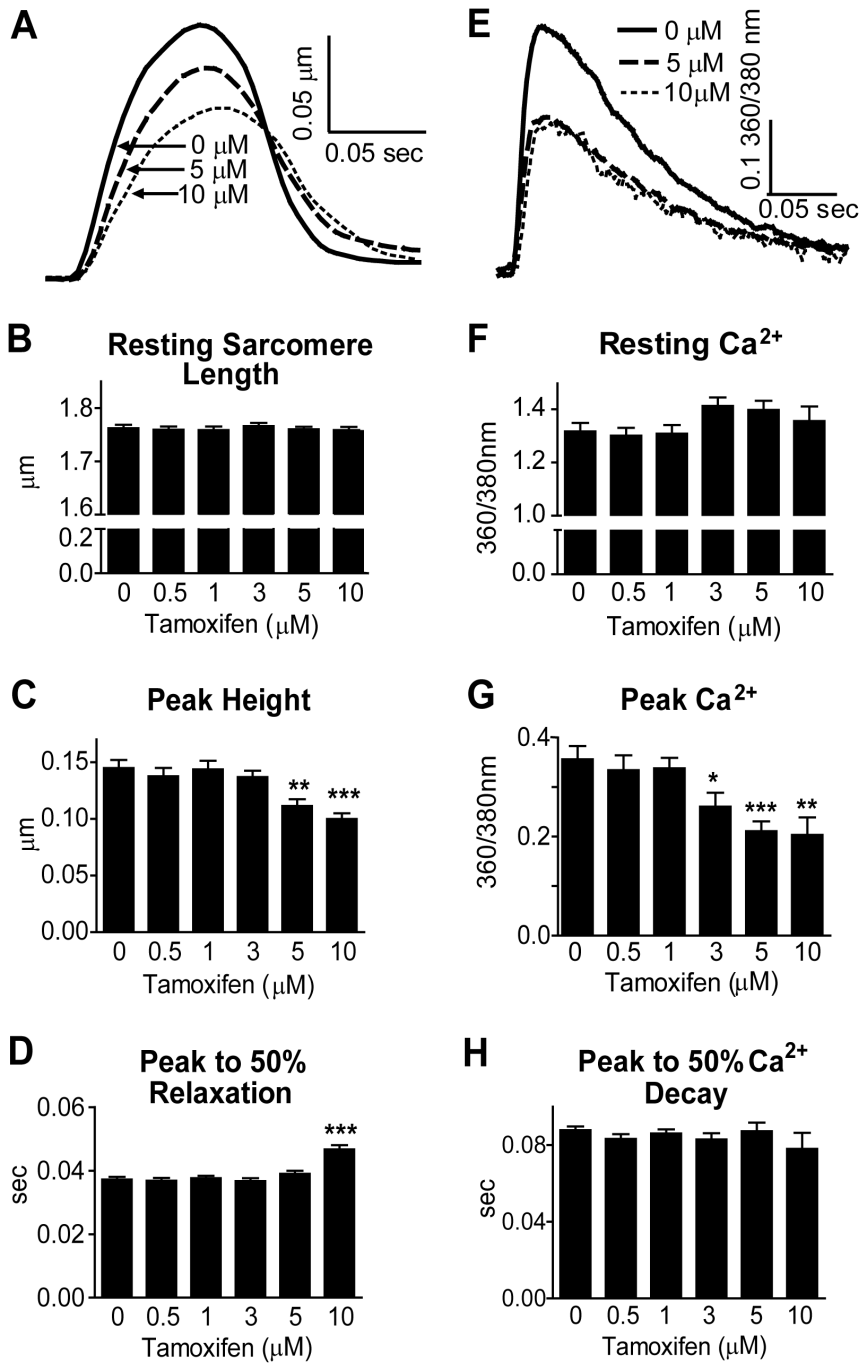
Sarcomere Length and Calcium Transient Measurements in Tamoxifen-Treated Rat Cardiac Myocytes. A) Representative traces of sarcomere shortening normalized to baseline. B-D) Sarcomere length data was collected from myocytes treated with Tam and paced at 0.2 Hz and 36 ± 1°C. Data are from four rat cardiac myocyte preparations, N = 46-52 cells/treatment. E) Representative traces of Ca^2+^ transients normalized to baseline. F-H) Myocytes were treated with Fura-2AM, and Ca^2+^ transient data was collected at 0.5 Hz and 36 ± 1°C after Tam treatment. Data are from three rat cardiac myocyte preparations, N = 11-30 cells/treatment. Data are presented as mean ± SEM. *P < 0.05, **P < 0.01, ***P < 0.001 compared to 0 µM.

**Figure 3:**
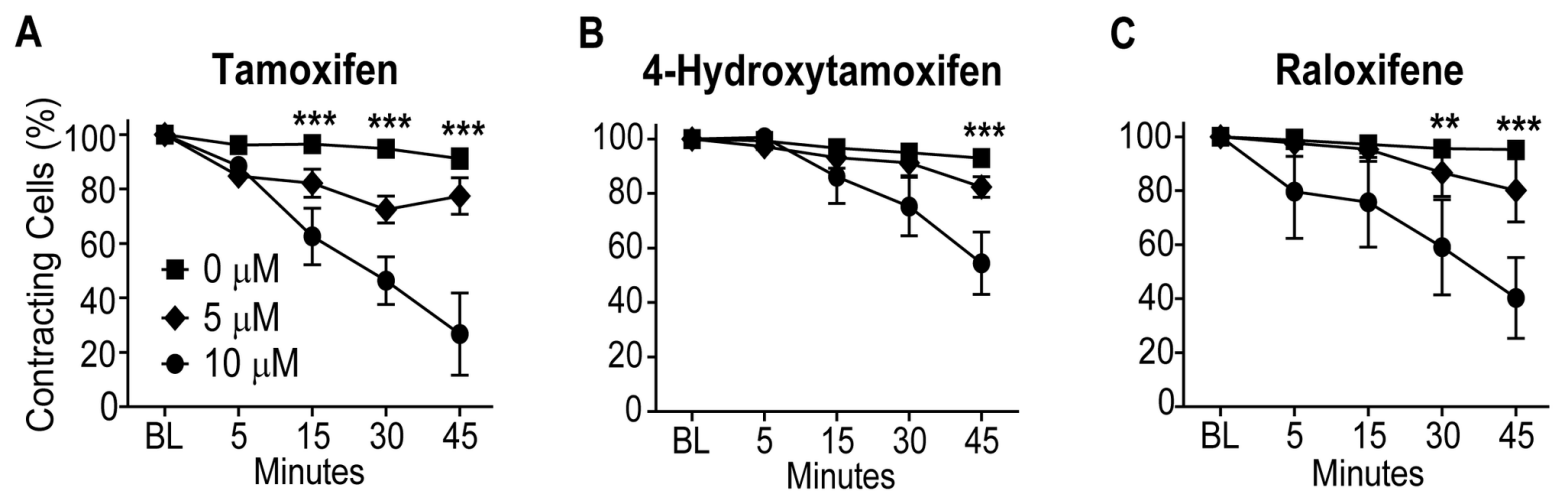
Stimulation-Induced Contractility with Tamoxifen, 4-Hydroxytamoxifen, and Raloxifene. Contracting cells in a field of view containing 30-50 cells treated with A) Tam, B) 4OHT, or C) Ral were counted over 45 minutes at 0.2 Hz. The percentage change from baseline in contracting cells at each time point was calculated, and data are presented as mean ± SEM, N = 3-4 counting experiments/treatment. **P < 0.01, ***P < 0.001 for 10 µM compared to 0 µM.

Tamoxifen had significant dose and time-dependent direct effects to alter the contractile function of isolated adult rat cardiac myocytes. Representative sarcomere length traces show reduction in the peak height of contraction and slowing in the rate of relaxation between the 0, 5, and 10 µM treatments (Figure 2A). Overall, resting sarcomere length was not altered (Figure 2B), while peak height decreased significantly with 5 and 10 µM Tam (Figure 2C), and the time from peak to 25%, 50% and 75% relaxation was increased with 10 µM Tam (Figure 2D, Table S1). There was a significant effect of 10 µM Tam to abolish stimulation-induced myocyte contraction beginning at 15 min after treatment. After 45 min of pacing at 0.2 Hz, there was >70% decrease in the percentage of visibly contracting rod-shaped myocytes with 10 μM Tam compared to vehicle (Figure 3A). 

To investigate the mechanism of acute Tam-induced myocyte contractile dysfunction, Ca^2+^ transients were measured. Peak height of the Ca^2+^ transient was decreased with 3, 5, and 10 μM Tam treatment (Figure 2E, G), closely reflecting the dose-dependent decrease in peak height of contraction (Figure 2A, C). Resting Ca^2+^ and Ca^2+^ decay time were not significantly altered by Tam, indicating Ca^2+^ reuptake is not a primary contributor to slowed sarcomere relaxation (Figure 2F, H, Table S1). 

### 4-Hydroxytamoxifen

**Figure 4:**
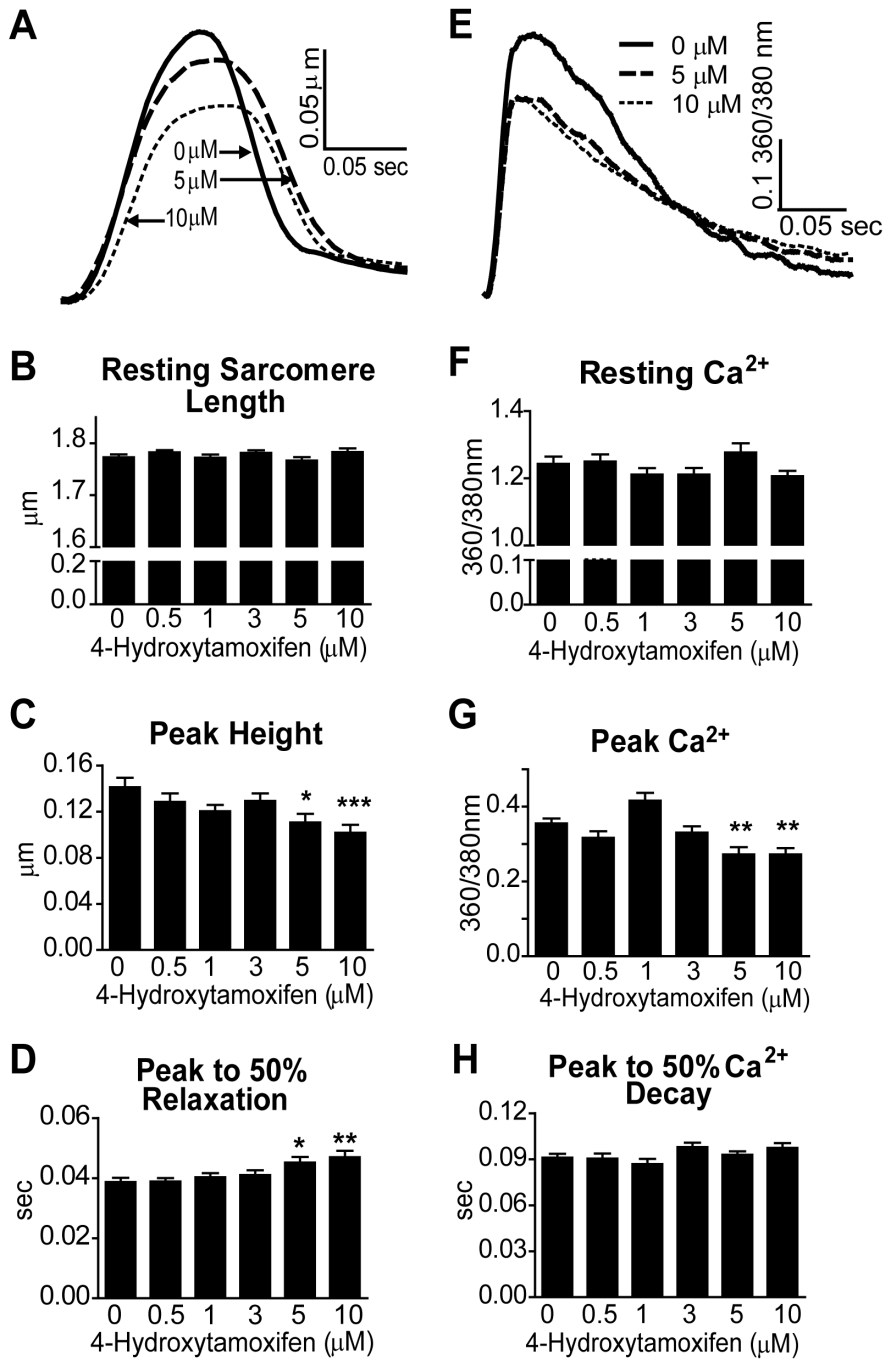
Sarcomere Length and Calcium Transient Measurements in 4-Hydroxytamoxifen-Treated Rat Cardiac Myocytes. A) Representative traces of sarcomere shortening normalized to baseline. B-D) Sarcomere length data was collected from myocytes treated with 4OHT and paced at 0.2 Hz and 36 ± 1°C. Data are from four rat cardiac myocyte preparations, N = 45-50 cells/treatment. E) Representative traces of calcium transients normalized to baseline. F-H) Myocytes were treated with Fura-2AM, and calcium transient data was collected at 0.5 Hz and 36 ± 1°C after 4OHT treatment. Data are from three rat cardiac myocyte preparations, N = 38-42 cells/treatment. Data are presented as mean ± SEM. *P < 0.05, **P < 0.01, ***P < 0.001 compared to 0 µM.

Sarcomere length and Ca^2+^ transient experiments were done using 4OHT (Figure 4) to investigate whether acute ER signaling has a role in the Tam-induced effects on cardiac myocytes. *In vivo*, Tam is hydroxylated by CYP2D6 in the cytochrome P450 pathway to yield the active metabolite 4OHT [31]. Estrogen receptors bind 4OHT with higher affinity than Tam, and 4OHT is approximately 100-fold more potent than Tam in the inhibition of cell growth in MCF-7 human breast cancer cells [32]. The hypothesis was that 4OHT will elicit more severe functional changes in cardiac myocytes than Tam if acute ER signaling is the major mechanism by which Tam inhibits contractility and Ca^2+^ transients. Results of experiments using 4OHT were similar to those of Tam treatment for sarcomere length (Figure 4A-D, Table S2), Ca^2+^ transients (Figure 4E-H, Table S2) and stimulation-induced contraction (Figure 3B). Because 4OHT is a more potent activator of ER than Tam, the similar functional effects of these two molecules suggest that the primary mechanism of action for the Tam-induced acute changes in Ca^2+^ and contractility is ER-independent. 

### Raloxifene

**Figure 5:**
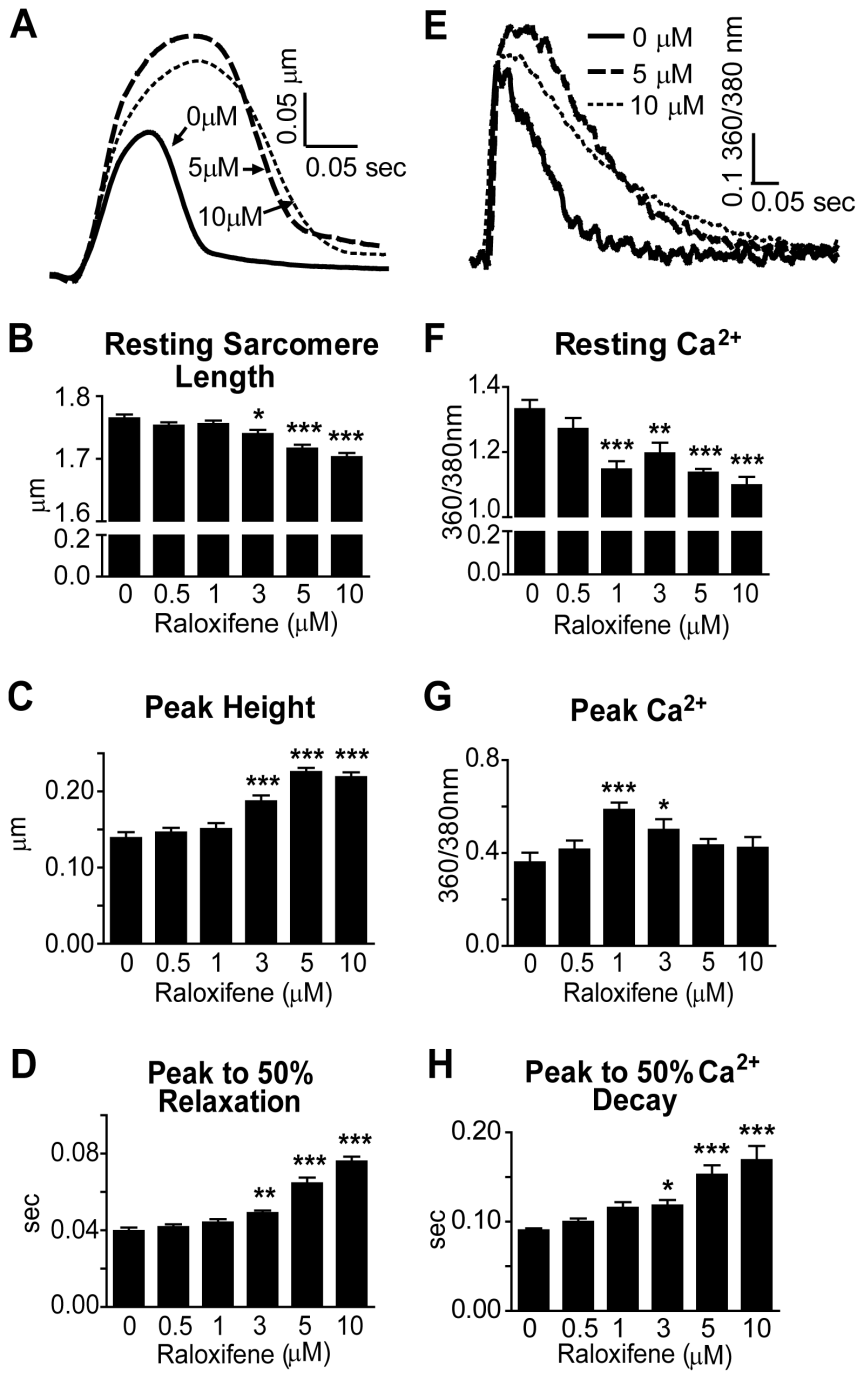
Sarcomere Length and Calcium Transient Measurements in Raloxifene-Treated Rat Cardiac Myocytes. A) Representative traces of sarcomere shortening normalized to baseline. B-D) Sarcomere length data was collected from myocytes treated with Ral and paced at 0.2 Hz and 36 ± 1°C. Data are from four rat cardiac myocyte preparations, N = 39-47 cells/treatment. E) Representative traces of Ca^2+^ transients normalized to baseline. F-H) Myocytes were treated with Fura-2AM, and Ca^2+^ transient data was collected at 0.5 Hz and 36 ± 1°C after Ral treatment. Data are from three rat cardiac myocyte preparations, N = 12-26 cells/treatment. Data are presented as mean ± SEM. *P < 0.05, **P < 0.01, ***P < 0.001 compared to 0 µM.

Ral is another SERM that has been used in place of Tam to address Tam-induced cardiomyopathy in Tg(αMHC-MerCreMer) mice [25]. In these experiments, higher doses of Ral and longer duration of treatment were required for effective gene knockdown, but cardiomyopathy was not apparent in these mice [25]. Unexpectedly, in isolated cardiac myocytes, acute application of Ral had significant effects on myocyte function that were distinct from Tam and 4OHT (Figure 5A, E). Ral markedly decreased resting sarcomere length and increased the peak height of contraction beginning at 3 μM (Figure 5B, C). In addition, Ral slowed the entire contraction/relaxation cycle, increasing the time to peak, and time from peak to 25%, 50% and 75% relaxation with 3-10 μM Ral (Figure 5D, Table S3). Ral at 10 μM abolished visible contraction in a higher percentage of myocytes compared to control after 30 min of stimulation (Figure 3C) and increased the incidence of after-depolarizations (data not shown) during pacing at 0.2 Hz. 

**Figure 6:**
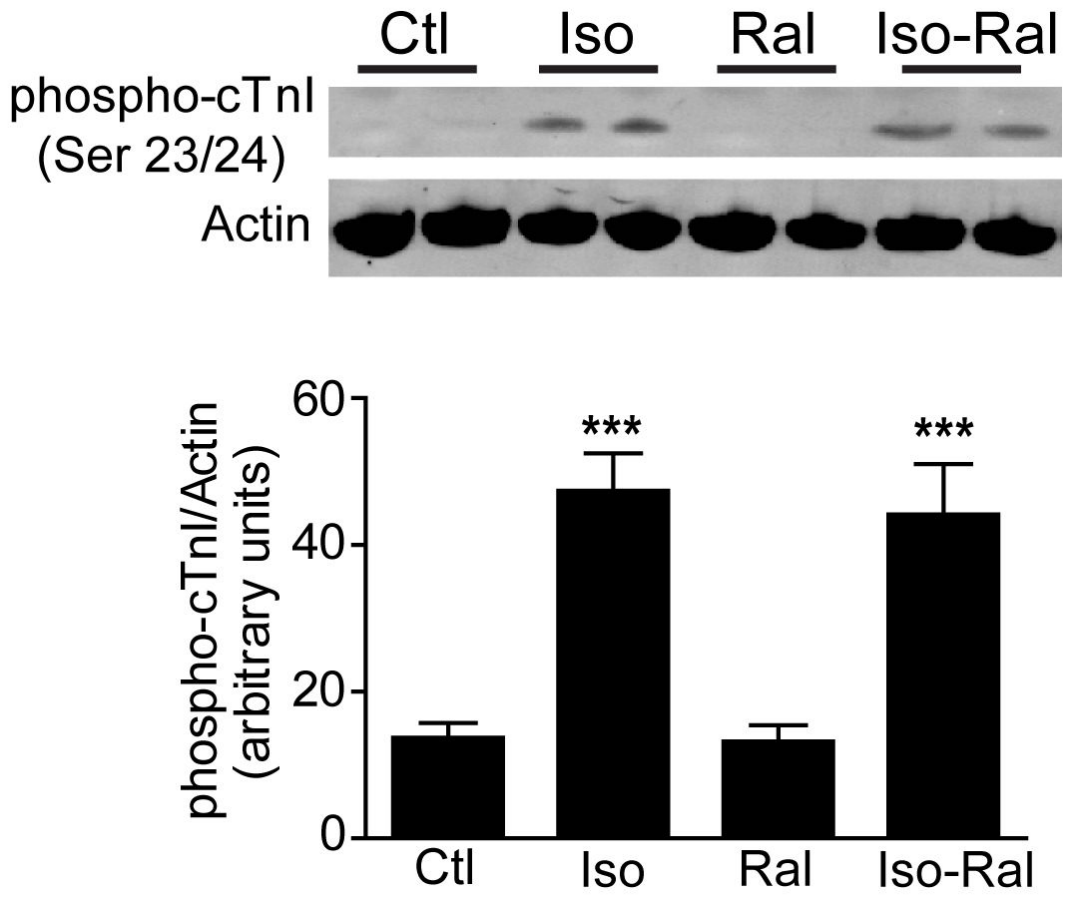
Phosphorylated cTnI with Raloxifene and Isoproterenol. Cells were treated with vehicle (Ctl), 10 nM isoprenaline (Iso), 10 µM raloxifene (Ral), or isoprenaline and raloxifene (Iso + Ral) for 15 min, after which cells were collected and lysed, protein concentration determined, and subjected to SDS-PAGE. Protein was transferred to PVDF membrane and probed for phospo-cTnI (Ser 23/24) and actin. The data are presented as mean ± SEM for the ratio of phospho-cTnI to actin. N = 14-18 coverslips of cells/group, ***P < 0.001.

Peak Ca^2+^ increased at 1 and 3 μM Ral and returned to control levels at 5 and 10 μM Ral (Figure 5G), in contrast to peak height of contraction, which dose-dependently increased (Figure 5C). Time to peak and times from peak to 25%, 50% and 75% Ca^2+^ transient decay were dose-dependently increased (Figure 5H and Table S3), reflecting sarcomere length measurements. A potential mechanism for slow Ca^2+^ transient decay is acute ER signaling through PKA to alter the phosphorylation status of cTnI [27,29,33]. To test this, cardiac myocytes were treated with Ral +/- isoproterenol to determine whether Ral causes dephosphorylation of cTnI. No changes in phosphorylation of cTnI were found, providing evidence that a mechanism independent of cTnI phosphorylation status is responsible for the effects of Ral to alter sarcomere shortening and Ca^2+^ transients (Figure 6).

### Tamoxifen and Raloxifene in Mouse Cardiac Myocytes

**Figure 7:**
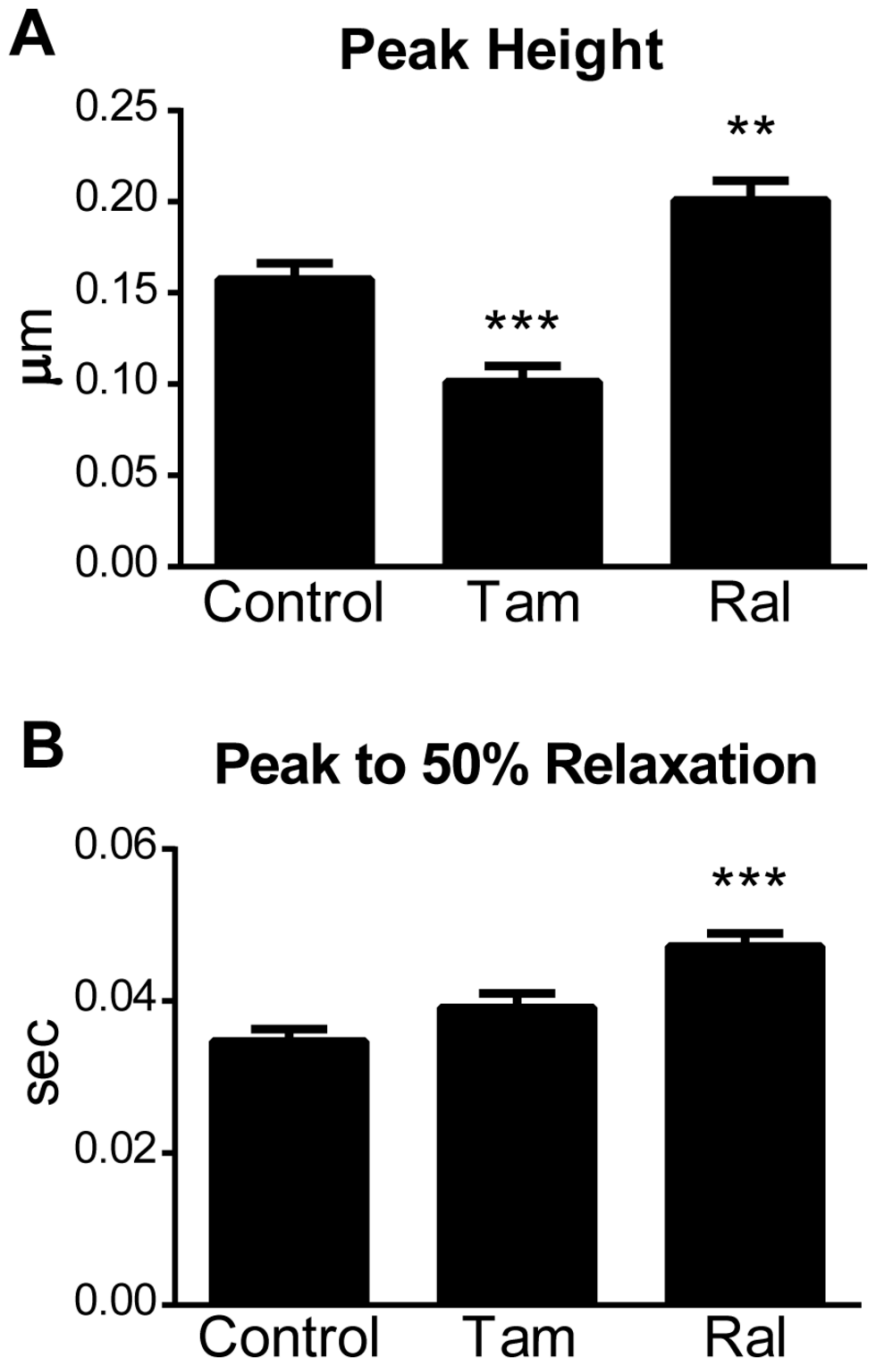
Sarcomere Length Measurements in Tamoxifen and Raloxifene-Treated Mouse Cardiac Myocytes. Sarcomere length data was collected from myocytes treated with 10 µM Tam or Ral and paced at 0.2 Hz and 36 ± 1°C. Data are from two mouse cardiac myocyte preparations, N = 21-25 cells/treatment. A) Peak height of contraction; B) Time from peak to 50% relaxation. Data are presented as mean ± SEM. **P < 0.01, ***P < 0.001 compared to 0 µM.

To determine whether Tam and Ral-induced changes in contractility were limited to rat myocytes or whether the effects occurred in other species, mouse myocytes were isolated and treated with 10 µM Tam or Ral. Similarly to rat, mouse cardiac myocytes exhibited a significant decrease in peak height of contraction with Tam treatment (Figure 7). Myocytes also stopped contracting over time, with a 75% reduction in contracting cells after 30 min with 10 μM Tam treatment. Although there was a trend towards increased relaxation time with Tam, this did not reach statistical significance. Mouse cardiac myocytes treated with Ral also exhibited similar changes in contractility when compared to rat myocytes. Specifically, at 10 μM Ral mouse myocytes had significantly increased peak height of contraction and time from peak to 50% relaxation (Figure 7). 

## Discussion

We determined the direct acute effects of Tam on cardiac myocyte contractile function to gain mechanistic insight into SERM-mediated cardiomyopathy. Main new findings here include the significant and differential effects of Tam, 4OHT, and Ral on cardiac myocyte contractility and Ca^2+^ handling. Tam and 4OHT decreased while Ral increased the peak height of contraction and this was accounted for by corresponding changes in peak Ca^2+^ transient amplitude. All SERMs tested had significant effects to slow myocyte relaxation. To aid discussion, a qualitative summary of the major results is presented in Table 1. These findings have implications for cardiac performance in gene excision studies and may have impact toward understanding cardiac effects in cancer patients on high-dose SERM treatment. 

**Table 1:** Summary of the effects of Tamoxifen, 4-hydroxytamoxifen and Raloxifene on cardiac myocyte function.

	Tamoxifen	4-hydroxytamoxifen	Raloxifene
Resting SL	↔	↔	↓↓
Resting Ca^2+^	↔	↔	↓↓
Time to Peak SL	↔	↔	↑↑
Time to Peak Ca^2+^	↑	↔	↑↑
Peak SL	↓	↓	↑↑
Peak Ca^2+^	↓↓	↓	↑*
SL Relaxation Time	↑	↑	↑↑
Ca^2+^ Transient Decay Time	↔	↔	↑↑

↑ increase; ↓ decrease; ↔ no change

* Increased at low concentrations but not high concentrations of Ral

Direct deleterious effects of Tam on myocyte contractile function are in keeping with the transient dilated cardiomyopathy reported in Tg(αMHC-MerCreMer) mice [25]. In that study, *in vivo* hemodynamics measurements provide evidence that Tam treatment contributed to the severe cardiac dysfunction, independent of the αMHC-MerCreMer transgene. In this work [25], there were significant differences in heart function, including heart rate, cardiac output, positive and negative derivatives, and tau, between mice 3 days and 3 weeks after discontinuation of Tam treatment. Our study does not preclude the synergistic effect of Tam plus MerCreMer to alter gene expression as highlighted in that study, but rather provides an additional mechanism by which Tam directly inhibits contractility and Ca^2+^ transients in an acute manner that cannot be explained by longer-term altered gene transcription. In addition, when Tam is removed from myocytes, function is partially restored (data not shown), in agreement with the transient nature of cardiomyopathy in Tam-treated Tg(αMHC-MerCreMer) mice. Although most of the experiments presented here utilized rat cardiac myocytes for experimental feasibility, mouse cardiac myocytes exhibited comparable functional deficiencies with Tam and Ral, increasing the relevance of our findings to Tam-treated mice in gene excision studies.

The data presented in this study give new insight into the mechanism of action for Tam to alter acute cardiac myocyte contractile function. Numerous studies have reported acute effects of ER activation in cardiac myocytes [28,29,34]. We hypothesized that 4OHT, which activates ERs with ~100-fold higher potency than Tam, would have amplified effects on cardiac myocyte function compared to Tam if acute, non-genomic signaling through ERs was responsible for its effects. Because the changes in contractility and Ca^2+^ handling were not different between Tam and 4OHT, ER-independent mechanisms likely had a primary role in the effects seen in our study [12,13,35]. The percentage of myocytes able to be stimulated in our study decreased significantly after only 15 min of 10 µM Tam treatment. The complete block of visible contractions over time with Tam may be the consequence of progressively decreased Ca^2+^ transient amplitudes as seen with Fura-2AM loading. Previous studies with Tam have reported diminished membrane potential through inhibition of both inward rectifier and outward delayed rectifier K^+^ currents and Na^+^ current, which can decrease action potential [12,13,35] and subsequently inhibit Ca^2+^ current through the LTCC [36]. Similar inhibition of LTCC current has been shown with supraphysiologic levels of 17β-estradiol [37]. Sufficiently diminished Ca^2+^ flux through the LTCC will fail to activate Ca^2+^-induced Ca^2+^ release and subsequently abolish contraction. 

Calcium transient decay time was not changed with Tam or 4OHT in our intact myocyte Ca^2+^ assay despite decreased sarcomere relaxation rate, suggesting a primary role for SERCA2a-independent mechanisms to slow relaxation in living myocytes treated with Tam. These data are in contrast to studies using isolated SR membranes or *in silico* computer modeling to test Tam effects. Tam inhibited Ca^2+^ uptake by SERCA2a in isolated SR vesicles [38]. Computer modeling revealed a potential Tam binding site in the transmembrane domain of SERCA2a near the thapsigargin binding site [39], suggesting the possibility for direct SERCA2a inhibition by Tam in this *in silico* analysis. Our data, in contrast, provide direct cellular evidence that Tam does not have an effect to modify SERCA2a activity to the extent that Ca^2+^ decay kinetics are affected. Mechanistically, this uncoupling of sarcomere relaxation from Ca^2+^ decay with both Tam and 4OHT suggests a direct interaction of Tam and 4OHT on the contractile apparatus, potentially resulting in Ca^2+^ sensitization. However, a limitation of our study is the different treatment and data collection timelines for the sarcomere length and Ca^2+^ transient experiments, preventing us from making a direct correlation between the two data sets. 

In contrast to Tam and 4OHT, Ral elicited a hypercontractile response and slowed the entire contraction/relaxation cycle. Most Ca^2+^ transient data mirrored sarcomere length data, suggesting that changes in Ca^2+^ flux are a primary mechanism driving mechanical changes in contraction and relaxation. Additionally, despite an initial increase in peak Ca^2+^ at 1 and 3 μM Ral, peak Ca^2+^ did not differ significantly from control at 5 and 10 μM Ral. These data point to Ral as a potential Ca^2+^ sensitizer of the myofilament at these high doses. Because Ral did not change phosphorylation of cTnI, ER-independent mechanisms appear to be contributing to the overall effects of Ral on myocyte functionality, similar to Tam and 4OHT. The net effect of Ral, however, may result from a combination of both ER-independent and ER-dependent mechanisms, which were not dissected in this study. The basis for the differential effects of Ral compared to Tam and 4OHT is not clear. Whereas Ral and Tam/4OHT are all SERMS, they have differing structures and affinities and this could underlie the divergent effects on myocyte function reported here.

Acute effects of Ral have been measured in guinea pig cardiac myocytes with the main findings being a decrease in Ca^2+^ current through the LTCC and decreased peak Ca^2+^ transient and contraction amplitude [40]. The mechanism was proposed to be an ER-dependent inhibition of the LTCC current. The divergent effects between this study and ours may partially be explained by the fundamental differences in Ca^2+^ handling in cardiac myocytes of guinea pigs compared to rats and mice, specifically with regard to Ca^2+^ flux between extracellular, intracellular, and SR compartments in diastole [41]. 

Typical Tam doses used clinically for the treatment and prevention of breast cancer are between 20 and 40 mg/day. An average dose of ~30 mg/day results in steady state serum concentrations of 0.1-0.6 µM [42], well beneath the concentrations causing functional impairment in our study. Much higher doses of Tam, between 120 and 720 mg/day, are used for experimental treatment of malignant gliomas [2–5] and multiple drug resistant cancers [6,7], with the goal of achieving serum concentrations of Tam and its metabolites between 3 μM and 10 μM. In some patients, these high doses of Tam caused LQTS [4,5,7], which can result in serious and sometimes fatal cardiac arrhythmias. Tam-induced LTQS is reversible when treatment is discontinued [4,10], which is in line with the partial functional recovery seen when Tam treatment is removed from cardiac myocytes (data not shown). Serum levels of Tam between 3 μM and 10 μM are within the range of concentrations causing significant deviations in myocyte contractility and calcium handling in our experiments.

To our knowledge, serum concentrations of Tam and its metabolites have not been measured in Tg(αMHC-MerCreMer) mice. High-dose Tam therapy in humans ranges from approximately 5-10 mg/kg/day, much lower than the 40-80 mg/kg/day dose given to Tg(αMHC-MerCreMer) mice. Although clearance of Tam from serum is faster in mice than humans, accumulation of Tam and its metabolites in tissues are significantly elevated 24 hours after 5 daily doses of 40 mg/kg [43], making Tam-induced effects on cardiac myocyte function a potential contributor to the transient dilated cardiomyopathy seen in the Tg(αMHC-MerCreMer) mouse model.

## Conclusions

In summary, the goal of the present study was to determine the effects of Tam, 4OHT, and Ral on mechanical function of cardiac myocytes. Of primary importance is the potential implications these results have both for patients taking high doses of Tam and for prudent utilization of Tam in the Tg(αMHC-MerCreMer) mouse model. We found significant deviations in contractile performance and Ca^2+^ handling resulting from acute treatment of cardiac myocytes with Tam, 4OHT, and Ral. Effects were seen primarily with 5-10 µM Tam and 4OHT, and 3-10 µM Ral. Because of the complexity of Tam’s effects on organismal physiology, the present study focused specifically on the acute and direct response in isolated cardiac myocytes. This approach allows for the measure of direct effects on individual myocytes, without confounding variables introduced *in vivo*. The results of this study have both clinical and experimental relevance. First, these data provide a functional link between studies showing action potential abnormalities and ion channel inhibition and clinical findings of LQTS with high-dose Tam. Second, our data showing decreased contractility accompanied by decreased calcium transient peak height provides a cell intrinsic direct mechanism for the Tam-induced dilated cardiomyopathy in Tg(αMHC-MerCreMer) mice that is independent of the αMHC-MerCreMer transgene. Mice given 40-80 mg/kg Tam for multiple days may obtain serum concentrations within the deleterious ranges found in the present study, making acute, non-genomic effects of Tam a likely contributor to the cardiac pathology found in this model. 

## Supporting Information

Table S1 **Sarcomere length and calcium transient measurements in Tamoxifen-treated cardiac myocytes.**
(PDF)

Table S2 **Sarcomere length and calcium transient measurements in 4-hydroxytamoxifen-treated cardiac myocytes.**
(PDF)

Table S3 **Sarcomere length and calcium transient measurements in Raloxifene-treated cardiac myocytes.**
(PDF)
