# Supplementary material for: Direct, Differential Effects of Tamoxifen, 4-Hydroxytamoxifen, and Raloxifene on Cardiac Myocyte Contractility and Calcium Handling
Source: PLoS One. 2013 Oct 24;8(10):e78768. doi: 10.1371/journal.pone.0078768 (PMC3811994; doi:10.1371/journal.pone.0078768)
Supplement: Table S1 — Sarcomere length and calcium transient measurements in Tamoxifen-treated cardiac myocytes. (PDF) [file pone.0078768.s001.pdf]

Table S1: Sarcomere length and calcium transient measurements in Tamoxifen-treated cardiac myocytes

Tamoxifen (μM)	0	0.5	1	3	5	10
Sarcomere Length						
**N**	47	46	49	47	52	50
**Departure Velocity (μm/sec)**	-3.616 ± 0.208	-3.569 ± 0.211	-3.630 ± 0.220	-3.449 ± 0.157	-2.747 ± 0.158**	-2.230 ± 0.120***
**Time to Peak (sec)**	0.075 ± 0.002	0.072 ± 0.002	0.073 ± 0.001	0.073 ± 0.001	0.072 ± 0.001	0.079 ± 0.036
**Return Velocity (μm/sec)**	3.256 ± 0.179	3.207 ± 0.215	3.259 ± 0.205	3.258 ± 0.183	2.247 ± 0.172**	1.872 ± 0.138***
**Peak to 25% Baseline (sec)**	0.025 ± 0.001	0.025 ± 0.001	0.026 ± 0.001	0.025 ± 0.001	0.026 ± 0.001	0.031 ± 0.001***
**Peak to 75% Baseline (sec)**	0.053 ± 0.001	0.053 ± 0.001	0.054 ± 0.001	0.052 ± 0.002	0.059 ± 0.002	0.070 ± 0.003***
Ca^{2+} Transients						
**N**	28	30	30	26	27	11
**Departure Velocity (μm/sec)**	48.00 ± 3.64	49.74 ± 4.72	49.99 ± 3.83	32.44 ± 3.23*	26.31 ± 2.58***	28.83 ± 6.56*
**Time to Peak (sec)**	0.023 ± 0.002	0.025 ± 0.002	0.027 ± 0.002	0.028 ± 0.002	0.028 ± 0.002	0.034 ± 0.004*
**Return Velocity (μm/sec)**	-3.086 ± 0.218	-2.809 ± 0.268	-2.864 ± 0.168	- 2.612 ± 0.343	-1.927 ± 0.161**	-2.111 ± 0.216
**Peak to 25% Decay (sec)**	0.050 ± 0.001	0.046 ± 0.001	0.049 ± -.001	0.048 ± 0.002	0.047 ± 0.002	0.042 ± 0.005
**Peak to 75% Decay (sec)**	0.143 ± 0.005	0.144 ± 0.006	0.147 ± 0.007	0.136 ± 0.007	0.143 ± 0.007	0.154 ± 0.023

N = number of total cells measured from 3-4 separate rat cardiac myocyte preparations.

*P < 0.05, **P < 0.01, ***P < 0.001 compared to 0 μM Tam.
