# Supplementary material for: Direct, Differential Effects of Tamoxifen, 4-Hydroxytamoxifen, and Raloxifene on Cardiac Myocyte Contractility and Calcium Handling
Source: PLoS One. 2013 Oct 24;8(10):e78768. doi: 10.1371/journal.pone.0078768 (PMC3811994; doi:10.1371/journal.pone.0078768)
Supplement: Table S2 — Sarcomere length and calcium transient measurements in 4-hydroxytamoxifen-treated cardiac myocytes. (PDF) [file pone.0078768.s002.pdf]

Table S2: Sarcomere length and calcium transient measurements in 4-hydroxytamoxifen-treated cardiac myocytes

**4OHT (µM)**	**0**	**0.5**	**1**	**3**	**5**	**10**
Sarcomere Length						
**N**	46	49	50	45	50	47
**Departure Velocity (µm/sec)**	-3.547 ± 0.221	-3.338 ± 0.235	-2.901 ± 0.142	-3.241 ± 0.200	-2.681 ± 0.210*	-2.436 ± 0.193***
**Time to Peak (sec)**	0.076 ± 0.003	0.074 ± 0.002	0.079 ± 0.002	0.077 ± 0.002	0.081 ± 0.002	0.081 ± 0.003
**Return Velocity (µm/sec)**	3.202 ± 0.222	2.978 ± 0.242	2.678 ± 0.161	2.841 ± 0.196	2.312 ± 0.217*	2.082 ± 0.202**
**Peak to 25% Baseline (sec)**	0.027 ± 0.001	0.026 ± 0.001	0.028 ± 0.001	0.028 ± 0.001	0.030 ± 0.001	0.032 ± 0.002**
**Peak to 75% Baseline (sec)**	0.055 ± 0.002	0.057 ± 0.003	0.057 ± 0.002	0.058 ± 0.003	0.066 ± 0.003*	0.066 ± 0.003*
Ca^{2+} Transients						
**N**	41	38	40	42	41	40
**Departure Velocity (µm/sec)**	46.03 ± 2.38	39.91 ± 56.14	56.14 ± 2.98*	41.07 ± 2.57	32.83 ± 2.50**	34.23 ± 2.42**
**Time to Peak (sec)**	0.026 ± 0.001	0.031 ± 0.002	0.029 ± 0.002	0.027 ± 0.002	0.028 ± 0.001	0.025 ± 0.001
**Return Velocity (µm/sec)**	-2.688 ± 0.125	-2.483 ± 0.179	-3.822 ± 0.381***	-2.287 ± 0.124	-2.048 ± 0.149	-1.942 ± 0.137*
**Peak to 25% Decay (sec)**	0.051 ± 0.001	0.052 ± 0.002	0.049 ± 0.001	0.054 ± 0.001	0.052 ± 0.002	0.052 ± 0.001
**Peak to 75% Decay (sec)**	0.156 ± 0.006	0.149 ± 0.006	0.151 ± 0.008	0.170 ± 0.007	0.155 ± 0.005	0.176 ± 0.008

N = number of total cells measured from 4 separate rat cardiac myocyte preparations.

*P < 0.05, **P < 0.01, ***P < 0.001 compared to 0 µM 4OHT.
