# Supplementary material for: Direct, Differential Effects of Tamoxifen, 4-Hydroxytamoxifen, and Raloxifene on Cardiac Myocyte Contractility and Calcium Handling
Source: PLoS One. 2013 Oct 24;8(10):e78768. doi: 10.1371/journal.pone.0078768 (PMC3811994; doi:10.1371/journal.pone.0078768)
Supplement: Table S3 — Sarcomere length and calcium transient measurements in Raloxifene-treated cardiac myocytes. (PDF) [file pone.0078768.s003.pdf]

Table S3: Sarcomere length and calcium transient measurements in Raloxifene-treated cardiac myocytes

**Raloxifene (μM)**	**0**	**0.5**	**1**	**3**	**5**	**10**
Sarcomere Length						
**N**	46	44	42	46	47	39
**Departure Velocity (μm/sec)**	-3.469 ± 0.237	-3.516 ± 0.173	-3.581 ± 0.230	-4.109 ± 0.207	-4.662 ± 0.172***	-4.133 ± 0.182
**Time to Peak (sec)**	0.078 ± 0.003	0.083 ± 0.003	0.086 ± 0.003	0.103 ± 0.003***	0.121 ± 0.005***	0.136 ± 0.005***
**Return Velocity (μm/sec)**	3.272 ± 0.239	3.230 ± 0.166	3.211 ± 0.203	3.599 ± 0.174	3.646 ± 0.146	2.843 ± 0.120
**Peak to 25% Baseline (sec)**	0.027 ± 0.001	0.030 ± 0.001	0.031 ± 0.001	0.035 ± 0.001**	0.048 ± 0.002***	0.055 ± 0.002***
**Peak to 75% Baseline (sec)**	0.056 ± 0.003	0.057 ± 0.002	0.059 ± 0.002	0.066 ± 0.002*	0.085 ± 0.004***	0.107 ± 0.004***
Ca^{2+} Transients						
**N**	26	22	23	24	26	12
**Departure Velocity (μm/sec)**	53.73 ± 6.48	56.68 ± 4.98	80.25 ± 5.87**	56.20 ± 6.33	45.62 ± 5.71	35.82 ± 6.34
**Time to Peak (sec)**	0.026 ± 0.002	0.027 ± 0.002	0.034 ± 0.003	0.051 ± 0.007***	0.057 ± 0.006***	0.052 ± 0.005**
**Return Velocity (μm/sec)**	-2.958 ± 0.317	-2.978 ± 0.223	-3.712 ± 0.302	-3.249 ± 0.323	-2.403 ± 0.254	-2.035 ± 0.329
**Peak to 25% Decay (sec)**	0.052 ± 0.002	0.056 ± 0.002	0.068 ± 0.004*	0.072 ± 0.005**	0.091 ± 0.006***	0.100 ± 0.008***
**Peak to 75% Decay (sec)**	0.142 ± 0.004	0.181 ± 0.009	0.198 ± 0.014*	0.203 ± 0.014*	0.274 ± 0.020***	0.292 ± 0.032***

N = number of total cells measured from 3-4 separate rat cardiac myocyte preparations.

*P < 0.05, **P < 0.01, ***P < 0.001 compared to 0 μM Ral.
